# Plasmonic Excitations of 1D Metal-Dielectric Interfaces in 2D Systems: 1D Surface Plasmon Polaritons

**DOI:** 10.1038/srep04536

**Published:** 2014-04-01

**Authors:** Daniel R. Mason, Sergey G. Menabde, Sunkyu Yu, Namkyoo Park

**Affiliations:** 1 Photonic Systems Laboratory, School of EECS, Seoul National University, Seoul 151-744, Korea

## Abstract

Surface plasmon-polariton (SPP) excitations of metal-dielectric interfaces are a fundamental light-matter interaction which has attracted interest as a route to spatial confinement of light far beyond that offered by conventional dielectric optical devices. Conventionally, SPPs have been studied in noble-metal structures, where the SPPs are intrinsically bound to a 2D metal-dielectric interface. Meanwhile, recent advances in the growth of hybrid 2D crystals, which comprise laterally connected domains of distinct atomically thin materials, provide the first realistic platform on which a 2D metal-dielectric system with a truly 1D metal-dielectric interface can be achieved. Here we show for the first time that 1D metal-dielectric interfaces support a fundamental 1D plasmonic mode (1DSPP) which exhibits cutoff behavior that provides dramatically improved light confinement in 2D systems. The 1DSPP constitutes a new basic category of plasmon as the missing 1D member of the plasmon family: 3D bulk plasmon, 2DSPP, *1DSPP*, and 0D localized SP.

Low-dimensional collective electron excitations at metal surfaces, the so-called surface plasmon-polaritons (SPPs), provide a route towards tremendous electric field enhancement^1^ and spatial confinement^1^, which can dramatically enhance light-matter interactions^2^. Conventionally, SPPs have been studied in low-dimensional noble-metal structures, where the SPP is intrinsically bound to a 2D metal-dielectric interface^1^. Although recently, graphene^3^, the atomically thin 2D hexagonal crystal of carbon atoms, has rapidly gained interest as a long-sought-after plasmonic material^4^,^5^,^6^,^7^,^8^,^9^,^10^,^11^,^12^,^13^,^14^,^15^,^16^,^17^,^18^,^19^,^20^,^21^ alternative to noble metals due to initial predictions^5^ of exceptional electric field confinement of the intrinsic 2D plasmonic excitations of doped graphene sheets^5^,^6^,^7^. Quasi-low-dimensional schemes aiming to further increase the electric field confinement of graphene plasmons, such as excitations of graphene ribbon structures^8^,^9^,^10^,^11^,^12^,^13^, p-n junctions^14^,^15^, discs^8^,^16^,^17^ and nanoresonators^18^, have been suggested.

Meanwhile, at the cutting edge of materials science are hybrid 2D crystals^22^,^23^,^24^,^25^, comprising laterally connected domains of distinct atomically thin materials, and which are mechanically continuous over macroscopic domains. Moreover, studies have shown that compositional transition can occur over atomic scale distances^22^,^23^,^24^,^25^ at a crystalline junction^25^ connecting the neighbouring domains. Efforts in this direction have been largely driven by prospects to further complement the outstanding properties of graphene^22^, and towards its implementation with other 2D materials such as the insulating hexagonal boron nitride (h-BN), for atomically thin electrical circuitry^23^,^24^. However, yet to be recognized is that hybrid 2D crystals further provide the first realistic platform on which a 2D metal-dielectric system with truly 1D metal-dielectric (1DMD) interface – that is, across which the sign of imaginary part of conductivity changes from positive (i.e., metallic response), to negative (i.e., dielectric response) – could be achieved and probed at optical frequencies. Although, while such a 1D interface is of remarkable fundamental simplicity, constituting the low-dimensional counterpart of the bulk metal-dielectric interface, the existence and nature of plasmonic excitations at 1DMD interfaces remain unknown.

In this study, we show that 1DMD interfaces in general 2D metal-dielectric systems support a fundamental and unique 1D plasmonic mode (1DSPP). Through an illustrative example on a hybrid graphene/graphene platform, we show how unique cutoff behaviour of 1DSPPs could allow for a dramatic improvement in the electric field confinement of plasmons in 2D systems, exceeding that of previously predicted quasi-1D and 1D plasmonic excitations in graphene^8^,^9^,^10^,^11^,^12^,^13^,^14^,^15^. In the considered example, we demonstrate confinement of the electric field intensity to modal areas over one million times smaller than the diffraction limit. Furthermore, from a fundamental perspective, the 1DSPP, as the fundamental excitation of a 1DMD interface, constitutes a new basic category of plasmon on its own: the missing 1D member of the plasmon family (3D bulk plasmon, 2DSPP, *1DSPP*, and 0D localised SP).

## Results

**Figure 1:**
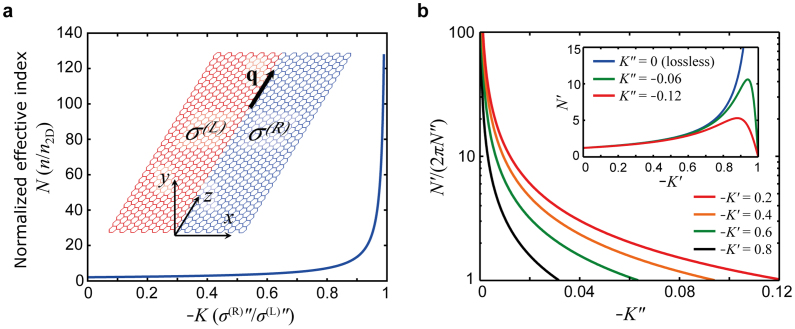
1DMD interface in a hybrid 2D system and dispersion of 1DSPPs. (a), Effective index *n* of the 1DMD interface plasmon (1DSPP) normalised to that of planar 2D plasmons in the metallic domain L (*n*_2D_) as a function of *K = σ*^(R)^″/*σ*^(L)^″ (*σ*^(L)^″ > 0; *σ*^(R)^″ < 0). Inset: Schematic of the considered 2D metal-dielectric system comprising two semi-infinite domains L (*x* < 0; metallic) and R (*x* > 0; dielectric) laterally connected along the *z* axis, with sheet conductivities of *σ*^(L)^ and *σ*^(R)^, respectively. We consider a plasmon with a wavevector **q** = *q***k** that propagates along and is localised to the 1D interface (coinciding with the *z* axis). (b), *K*″ dependence of *N*′/(2π*N*″) at indicated values of *K*′. Inset: lossy dispersion of *N*′ for example values of loss *K*″ = −0.06 (green), *K*″ = −0.12 (red), as compared to lossless dispersion (blue).

We start by considering a 2D metal-dielectric (MD) system in the *y* = 0 plane consisting of two semi-infinite domains that are laterally connected along the *z* axis with sheet conductivities of *σ*^(L)^ = *σ*^(L)^′ + *iσ*^(L)^″ (*x* < 0) and *σ*^(R)^ = *σ*^(R)^′ + *iσ*^(R)^″ (*x* > 0) (see inset in Fig. 1a) and immersed in a uniform dielectric with relative dielectric permittivity *ε*. Without loss of generality, we take regions L and R as the metallic (*σ*^(L)^″ > 0) and dielectric (*σ*^(R)^″ < 0) domains, respectively. At this point, to simplify our discussion, we omit ohmic and interband losses in both domains (*σ*^(R,L)^′ = 0; the lossy case is considered below and in the Supplementary Information). According to the theory of Volkov and Mikhailov^26^,^27^, the dispersion relation of plasmons propagating along the 1DMD interface (*z* = 0) in the quasi-static limit can be shown to be (see Methods): 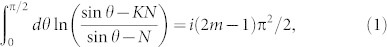
where *σ*^(R)^″ = *Kσ*^(L)^″ (*K* < 0), the normalised effective index *N* = *n*/*n*_2D_ (*N* > 0; *n = q/k*_0_; *k*_0_ = *ω*/*c*) is the effective index of the 1D interface plasmon with wavenumber *q* normalised to that of the planar 2D transverse magnetic (TM) plasmons of region L, *n*_2D_ = 2*cεε*_0_/*σ*^(L)^″ (Ref. 5), and *m* is an integer.

While equation (1) lacks a closed-form solution, our numerical solution indicates (see Methods) a dispersion relation of the form 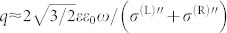, and *N*(*K*) is plotted in Fig. 1a. We find that non-leaky plasmons (i.e., *N* is purely real) exist strictly in the window 0 ≤ −*K* < 1. At *K* = 0, the plasmon wavenumber takes its minimum value as that of the bare edge plasmon^21^,^28^ 
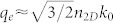
. As |*K*| → 1, that is, when the electric susceptibilities on the two sides of the interface become equal (|*σ*^(R)^″| → *σ*^(L)^″), the plasmon wave number diverges. Interestingly, this divergent behaviour reveals a strong connection to the conventional 2D surface plasmon polaritons (SPPs) localised to bulk metal-dielectric interfaces^29^. Indeed, in the absence of ohmic loss, the wavenumber of the SPPs (*q*_SPP_ = *n*_SPP_*k*_0_) diverges at the surface plasmon (SP) frequency *ω*_SP_ given by the non-retarded SP condition^29^: *ε*_d_ + *ε*_m_(*ω*_SP_) = 0; *ε*_d_ > 0 and *ε*_m_ < 0 are the relative permittivities of the dielectric and the metal, respectively. Likewise, at the 1DMD interface, we note the cutoff condition *K* = −1, or *σ*^(R)^″ + *σ*^(L)^″ = 0 (*σ*^(R,L)^′ = 0). This reveals that the plasmonic mode of a 1DMD interface is a *1D manifestation of SPPs* *in 2D systems* (1DSPP). An important consequence of the reduced dimensionality can be observed in the respective divergence behaviours: the SPP behaviour takes the form 
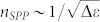
 (Δ*ε* = *ε*_d_ − |*ε*_m_|), which diverges much less rapidly than that of the 1DSPP, *n*~1/Δ*σ* (Δ*σ* = *σ*^(L)^″ − |*σ*^(R)^″|).

We note that in the earlier works of Volkov and Mikhailov^26^,^27^ they considered only conventional 2D electron gases (2DEG) with *σ*^ (R,L)^″ > 0 i.e., where both domains (L and R) are metallic. In that case, while there is no solution to equation (1) for purely real *N*, there is a solution for complex *N* which corresponds to a so-called inter-edge plasmon that is damped as it leaks into the extended 2D plasmons supported by the domain with the smaller (positive) imaginary part of conductivity^27^. In contrast, the 1DSPP (existing only in the 2D metal-dielectric system when 0 ≤ −*K* < 1) is entirely non-leaky, and completely bound to the 1DMD interface. It is also important to note a clear distinction between the 1DSPP, and the recently predicted quasi-1D plasmonic excitations of graphene p-n junctions^14^,^15^, according to fundamental differences between their respective physical origins. The multiple plasmon modes of a graphene p-n junction^14^,^15^ owe their manifestation to a spatial gradient of the imaginary part of conductivity (i.e., *σ*″), much like the multiple modes of a graded-index waveguide, and the graphene is everywhere metallic. In contrast, the 1DSPP manifests exclusively as a consequence of the metallic-to-dielectric transition across the 1DMD interface, either side of which the conductivity is spatially uniform. In this regard, we predict that the IR edge wave observed graphically in Vakil et al. (Ref. 9) is in fact due to the excitation of 1DSPPs, rather than p-n junction plasmons as suggested therein.

The lossy 1DSPP dispersion (i.e., when either *σ*^(L)^′, *σ*^(R)^′ ≠ 0) is readily obtained on introducing the complex parameter *K* defined in terms of the complex conductivities *K* = *σ*^(R)^/*σ*^(L)^ = *K*′ + *iK*″, and solving for the complex normalised effective index *N* = *n*/*n*_2D_ = *N*′ + *iN*″ (where *n*_2D_ = *i*2*cε_0_*/*σ*^(L)^ – Ref. 5). In the limit that the loss is wholly confined to the dielectric domain (*σ*^(L)^′ = 0, *σ*^(R)^′ ≠ 0), we obtain the convenient and meaningful expressions *K*′ = *σ*^(R)^″/*σ*^(L)^″ (i.e., same as the lossless case), and *K*″ = −*σ*^(R)^′/*σ*^(L)^″. Thus we may imagine that fixing *K*′ and simultaneously increasing |*K*″| corresponds to fixing of the imaginary part of conductivity of the L and R domains, and linearly increasing the loss (*σ*^(R)^′) in the dielectric domain. Conversely, fixing |*K*″| and increasing *K*′ is equivalent to fixing *σ*^(R)^′ and linearly increasing *σ*^(R)^″. Meanwhile, the quantity *N*′/(2π*N*″) reduces to the propagation length defined as the number of optical cycles within one exponential decay length (l = Re(*q*)/[2*π*Im(*q*)]). We note that this physical scenario is particularly relevant to a hybrid graphene/graphene metal-dielectric system at finite temperature (see Supplementary Information) where the dielectric response of domain R is accompanied by interband losses which strongly dominate the overall loss (i.e., relative to ohmic loss in either domain).

The green and red curves in the inset of Fig. 1b show *N*′ when loss is introduced through two example values *K*″ = −0.06 and *K*″ = −0.12. Similarly to the behavior of conventional SPPs on a lossy metal surface^29^, the loss is shown to destroy the divergent cutoff behavior of the 1DSPP owing to heavy damping, the onset of which occurs in some vicinity of *K*′ that is specific to *K*″ (compare where *N*′ begins to decrease at the two values of *K*″). To illustrate and quantify the onset of 1DSPP damping, the main window of Fig. 1b shows the *K*″ dependence of the 1DSPP propagation length at several fixed values of *K*′. At a given value of *K*″, the propagation length decreases with an increase in *K*′, which is typical of plasmons as their wave number increases (recalling that *q* increases with *K*′). We note that, at each value of *K*′, the curve is cutoff at the x-axis corresponding to the propagation length of just one wavelength (i.e., a strongly damped 1DSPP). It is illustrative to compare the *K*″ cutoff with a particular *K*′ e.g., when *K*′ = −0.6, the cutoff is *K*″ ~ −0.06, corresponding to *K*″/*K*′ = −*σ*^(R)^′/*σ*^(R)^″ ~ 0.1. Obviously, the loss is more forgiving for smaller *K*′, e.g. when *K*′ = −0.2, the cutoff is *K*″ ~ −0.12, corresponding to −*σ*^(R)^′/*σ*^(R)^″ ~ 0.6.

Now we present an illustrative and topical example of 1DSPPs in a hybrid graphene/graphene metal-dielectric system, in which the random phase approximation (RPA) provides convenient closed-form expressions for the optical conductivity in both domains^5^,^6^,^7^,^30^. The 1DMD interface is achieved by appropriate doping of both graphene domains; in the local and zero-temperature limit considered here (see Ref. 30 and Methods), the doping would correspond to 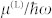 > 0.6 and 0.5 < 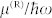 < 0.6, where *μ*^(L,R)^ is the Fermi energy of domain L or R, respectively, and details at finite temperatures are given in the Supplementary Information. Our numerical calculations suggest that the existence of 1DSPPs is not critically dependent on an abrupt sign change of *σ*″ at the interface (see Supplementary Information), so hybrid graphene systems that support 1DSPPs may also be realised by proposed electrical gating^9^ or substrate controlled^31^ schemes for spatially non-uniform graphene doping in addition to patterned growth^22^,^23^,^24^,^25^. We note that in an electrical gating scheme^9^, the absence of a well-defined edge separating the metallic and dielectric graphene domains would reduce edge effects such as those associated with zigzag edges in graphene and known to be responsible for strong plasmon damping (see for example Ref. 32).

**Figure 2:**
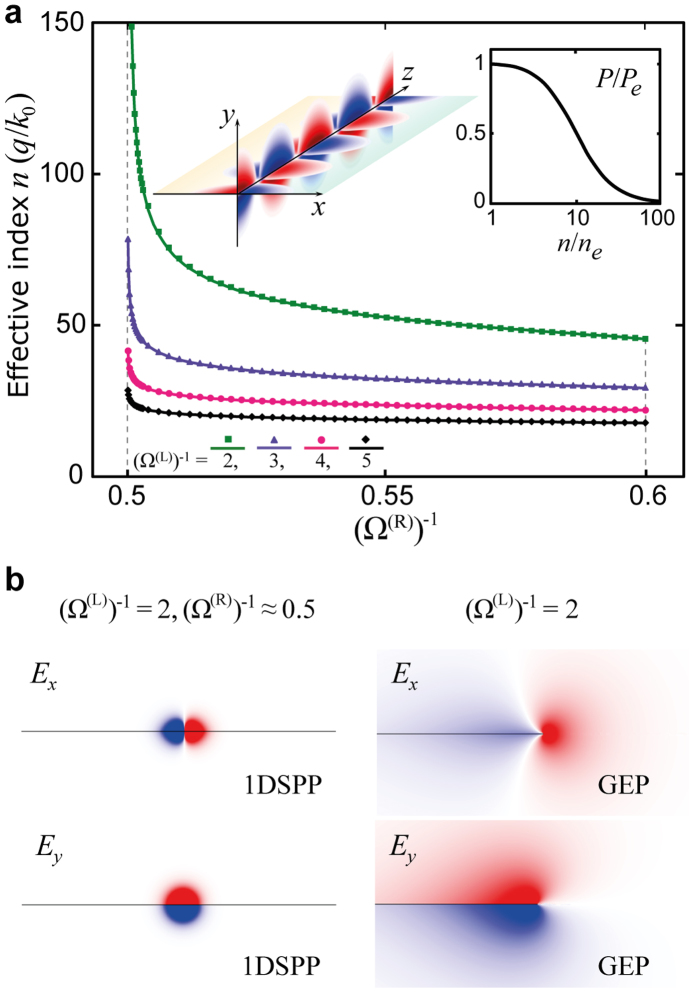
1DSPPs in a graphene/graphene hybrid 2D system. (a), Effective index *n* = *q*/*k*_0_ of the 1DSPP on a sample hybrid graphene/graphene system as a function of 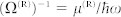; the different curves correspond to 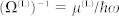 = 2, 3, 4, and 5. Excellent agreement is observed between the analytic dispersion equation (1) (solid curves) and FEM simulations (data points). Left inset: Spatial evolution of the 1DSPP electric field components *E_x_* (*xz* slice) and *E_y_* (*yz* slice); (Ω^(L)^)^−1^ = 2 and (Ω^(R)^)^−1^ ≈ 0.5. Right inset: Dependence of the net power flow *P* (along *z*) on the effective index *n* of the 1DSPP, normalised to that of the graphene bare edge plasmon (*P*_e_, *n*_e_). (b), Electric field components of the 1DSPP for the indicated parameters; the profiles on the left correspond to the 1DSPP near the cutoff, while those on the right correspond to the graphene bare edge plasmon (GEP) [the horizontal line indicates the graphene plane; the red (blue) colour denotes a positive (negative) sign].

The conductivities on either side of the interface are completely determined by the respective normalised frequencies^30^ 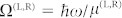. As it has been shown that the dispersion of 1DSPPs (relative to *n*_2D_) is completely determined by the dimensionless conductivity *K*, this behaviour suggests a flexible tunability of *n* through the variation of either *σ*^(R)^″ (*μ*^(R)^) or *σ*^(L)^″ (*μ*^(L)^) with the doping of either graphene domain. We demonstrate this tunability and simultaneously verify our quasi-static analytical results by employing a mode-solver tool in the numerical finite element method (FEM) package COMSOL, which includes retardation effects. In Fig. 2a, we show the calculated dependence of *n* on *μ*^(R)^ for several fixed values of *μ*^(L)^ (see respective curves) and the spatial evolution of the 1DSPP electric field components *E_x_* and *E_y_*. Note the normalisation of *μ*^(R,L)^ in terms of 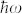; this was verified by comparing all results at the frequencies *f* = 20 THz and 80 THz. One can observe excellent agreement between the dispersion analytically predicted by equation (1) (solid curves) and the numerically determined data points. The monotonic increase in *n* as *μ*^(L)^ decreases (at fixed *μ*^(R)^) can be readily understood in terms of the corresponding decrease in *σ*^(L)^″and thus Δ*σ* (recalling that *n* ~ 1/Δ*σ*). Each curve diverges asymptotically towards a cutoff value of *μ*^(R)^ given by the condition *σ*^(L)^(*μ*^(L)^) + *σ*^(R)^(*μ*^(R)^) = 0. The cutoff value of *μ*^(R)^ approaches 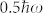 as *μ*^(L)^ increases. When the plasmon energy 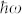 and the Fermi energy *μ*^(L)^ are of approximately the same order of magnitude (corresponding to the typical practical situation), the cutoff is very close to 0.5 because of the relative strengths of the interband and intraband terms in the graphene conductivity (see Methods).

The electric field profiles in Fig. 2b combined with the plot of the net power flow (inset in Fig. 2a; normalised to the graphene bare edge plasmon^21^ (GEP), i.e., when *σ*^(R)^ = 0) demonstrate the nature of the cutoff dynamics at the 1DMD interface. The net power flow parallel to the interface is given by the integral of *S_z_* = E_x_H_y_* − E_y_H_x_* over the *xy* plane. *S_z_* is symmetric about the *x* axis, although the terms E_x_H_y_* and E_y_H_x_* both exhibit odd sign parity about the *y* axis; thus, cumulative integration on either side of the interface leads to their respective partial cancellation. This cancellation is weak in the case of GEPs because of the strong field asymmetry, and therefore, the net power flow is significant. Conversely, as the cutoff condition (*σ*^(R)^″ → −*σ*^(L)^″) is approached, the symmetrisation of the field components caused by equal charge screening on either side of the interface (analogous to conventional SPPs near the surface plasmon frequency^29^) leads to a strong reduction in the net power flow as the group velocity approaches zero.

**Figure 3:**
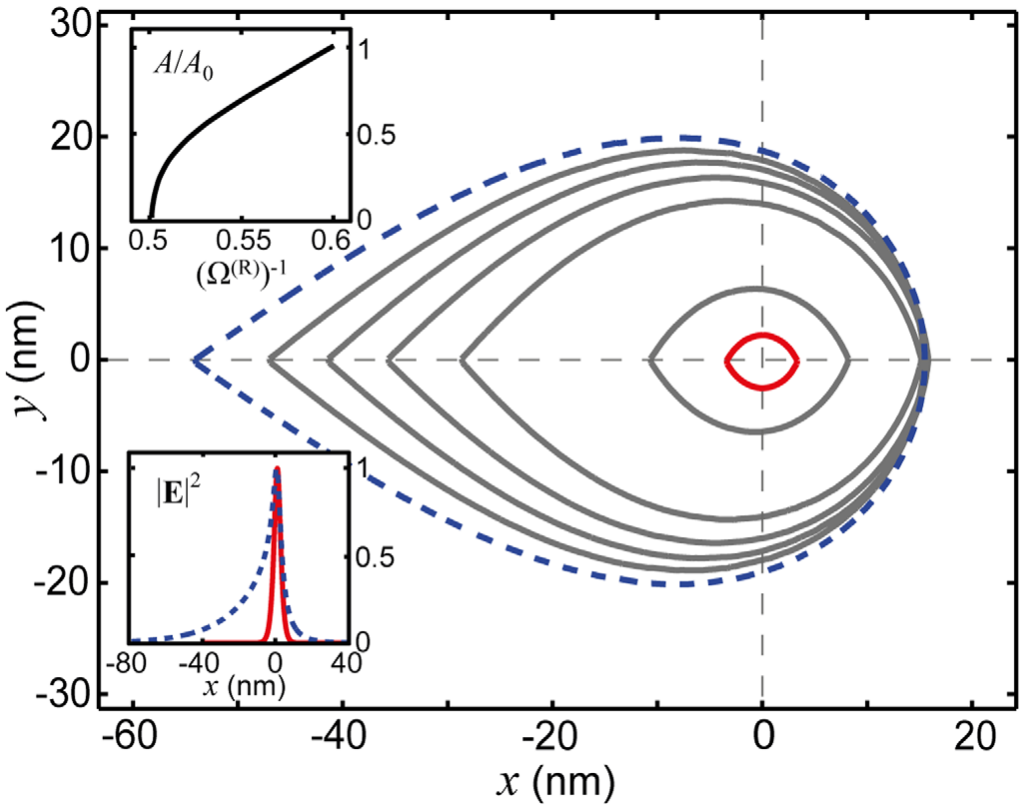
Enhanced modal confinement of 1DSPPs. Mode cross section of 1DSPPs as the cutoff condition is approached (blue-dashed *K* = 0 → red *K* = −0.87). Concentric contours, from the outermost to the innermost contour, correspond to (Ω^(R)^)^−1^ = 0.6, 0.58, 0.56, 0.54, 0.52, 0.501, and 0.5002. The blue-dashed contour exactly coincides with that of the graphene bare edge plasmon (GEP). Each contour is defined as a line |**E**|^2^ = constant enclosing an area *A* (in the *xy* plane), such that 
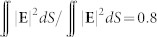
. Insets: (top) Mode cross section area *A* normalised to that of the GEP *A*_0_ as a function of (Ω^(R)^)^−1^ and (bottom) normalised intensity of the electric field (2 nm above the graphene) for the extreme cases (Ω^(R)^)^−1^ = 0.6 (blue-dashed) and (Ω^(R)^)^−1^ = 0.5002 (red). *f* = 50 THz; (Ω^(L)^)^−1^ = 2.

Figure 3 shows the evolution of the 1DSPP mode cross section (see caption for details) as the cutoff is approached, in comparison with the GEP. We note the tremendous localisation of the electric field intensity (lower inset) to regions from *A_0_* ≈ 5 × 10^−5^λ_0_^2^ to *A* ≈ 6 × 10^−7^λ_0_^2^ as |*K*| increases in the range 0 ≤ |*K*| ≤ 0.87. Thus, the spatial confinement of the electric field intensity of the 1DSPP in the hybrid graphene/graphene system is on the order of one million times smaller than the diffraction limit (~λ_0_^2^; λ_0_ is the vacuum wavelength); a two-order-of-magnitude improvement over the graphene bare edge plasmon (i.e., when *K* = 0).

## Discussion

We emphasise that the above results are not limited to the presented example of graphene but are generalizable to any isolated 1DMD interface in a hybrid 2D system characterised by the same value of *K*. 1DMD interfaces in graphene/graphene hybrid 2D systems at finite temperature are intrinsically lossy because of the presence of interband transitions, which are essential to achieving the dielectric character in one domain (i.e., *σ*^(R)^″ < 0; *K* < 0), and further exhibit a temperature-dependent upper limit on |*σ*^(R)^″| (and |*K*|). Nevertheless, we predict the propagation lengths of 1DSPPs on hybrid graphene systems to be several plasmon wavelengths at room temperature (*T* = 300 K) and ~100 plasmon wavelengths at the temperature of liquid nitrogen (*T* = 80 K); for example, at a free space wavelength λ = 1.8 μm we find *l* ~100nm (*μ*^(L)^ = 1 eV, *μ*^(R)^ = 0.42 eV, *T* = 300 K) and *l* ~ 2.6 μm (*μ*^(L)^ = 1 eV, *μ*^(R)^ = 0.40 eV, *T* = 80 K), respectively (see Supplementary Information for full details). On the other hand, graphene/h-BN hybrid structures are promising systems for supporting 1DSPPs with large propagation lengths. Because of the dielectric character of h-BN, one could realise a graphene/h-BN 1DMD interface with separate tunability of the graphene conductivity in real time using external gating, and the plasmon propagation length in this system would be limited only by the small ohmic losses in high-mobility graphene. Although in the case of an atomically sharp transition between neighbouring domains in hybrid 2D crystals, consideration of edge related effects (such as electronic edge states^32^) would be important to accurately determine the 1DSPP dispersion and loss properties.

In summary, we have predicted the existence of a fundamental 1D plasmonic mode of 1D metal-dielectric interfaces in 2D systems (1DSPP). The effective index of 1DSPPs diverges asymptotically towards a cutoff as the magnitudes of the susceptibilities on the two sides of the interface become equal, in striking analogy to conventional SPPs at bulk metal-dielectric interfaces. On a sample 2D metal-dielectric graphene/graphene platform, highly sensitive tunability of the 1DSPP dispersion was demonstrated via the doping of either graphene domain, which further allowed for the achievement of spatial confinement of electric-field intensity to regions orders of magnitude smaller than that of the plasmonic excitations of a bare graphene edge (GEP). The unique and tuneable cutoff behaviour of 1DSPPs thus presents a means for dramatic enhancement of light confinement in 2D systems over other 1D or quasi-1D excitations, such as GEPs or plasmonic excitations of graphene p-n junctions. At a frequency of 50 THz, we predicted tremendous localisation of the electric-field intensity of the 1DSPP to a modal area more than one million times smaller than the diffraction limit. As a new member of the existing plasmon family of bulk plasmons, surface plasmons, localised plasmons, etc., we foresee a new field of low-dimensional plasmonics based on 1DSPPs, particularly branching out into various multi-material 2D systems^22^,^23^,^24^,^25^.

## Methods

### 1DSPP Dispersion: Derivation and Analysis

We consider a hybrid 2D system in the *y* = 0 plane comprising two semi-infinite domains laterally connected along the *z* axis with sheet conductivities of *σ*^(L)^ = *σ*^(L)^′ + *iσ*^(L)^″ (*x* < 0) and *σ*^(R)^ = *σ*^(R)^′ + *iσ*^(R)^″ (*x* > 0) (see inset in Fig. 1) immersed in a uniform medium with relative dielectric permittivity *ε*. In the quasi-static theory of Volkov and Mikhailov^26^,^27^, based on the solution to Poisson's equation with an assumed electric potential of the form *φ*(**r**) = *φ*_0_(*x*,*y*)exp(*iq*_z_z−*iωt*), the dispersion relation of plasmons propagating along a 1D junction between two adjoining 2D electron gases (2DEG) is given by^26^

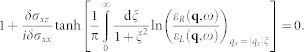


**Figure 4:**
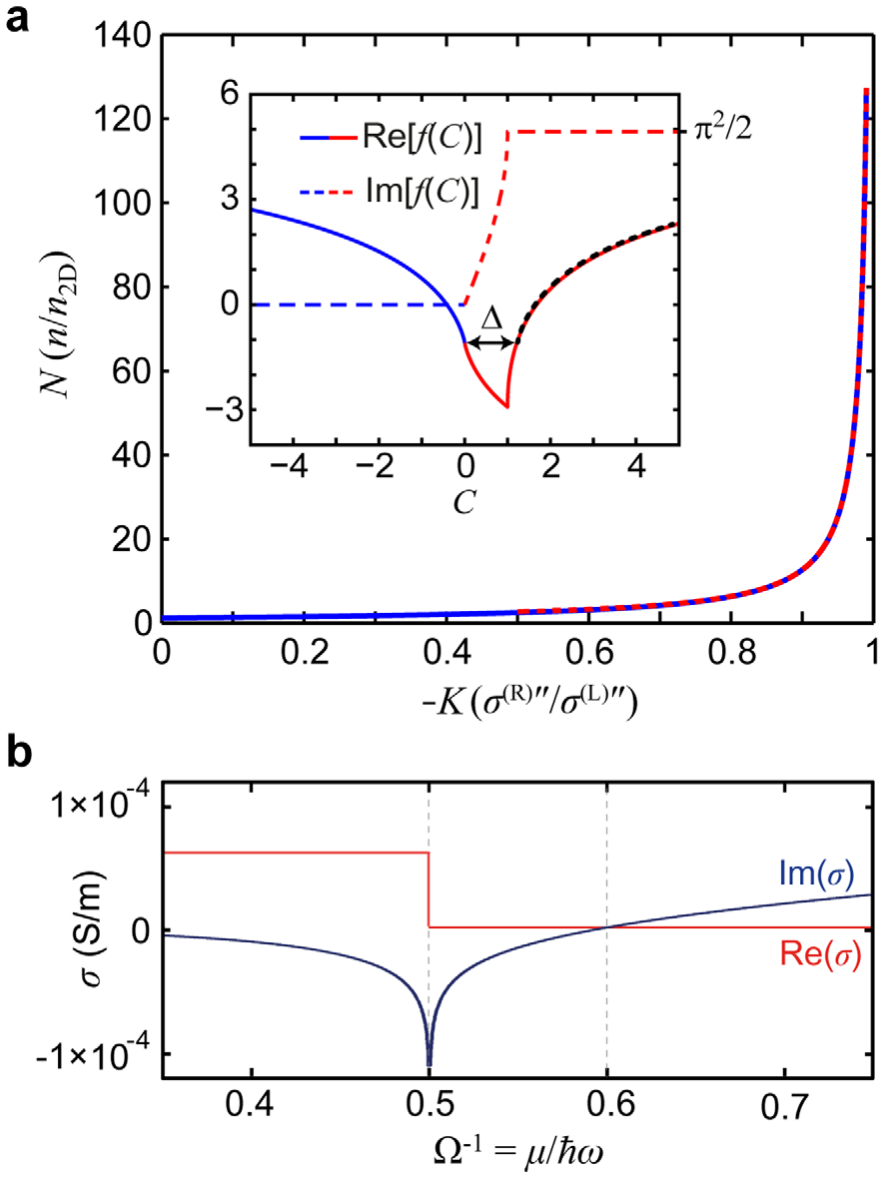
1DSPP dispersion and graphene conductivity. (a), The numerical solution [solid blue curve; equation (1)] and the asymptotic expression [dashed red curve; equation (2) for |*K*| > 0.5] are both shown. Inset: Real (solid curve) and imaginary (dashed curve) parts of the function *f*(*C*). The colour distinguishes *C* > 0 (red) from *C* < 0 (blue). The black overlaid dotted curve corresponds to Re[*f*(*C* + Δ)] ≈ Re[*f*(−*C*)]. (b), Conductivity of graphene in the local and zero-temperature limit.

Here, *δσ_βγ_* = *σ*^(R)^*_βγ_* − *σ*^(L)^*_βγ_*, where *β* and *γ* are tensor indices (*x*, *z*), and *ε_R,L_* are the effective dielectric permittivities of the left (L) and right (R) 2DEG^26^; 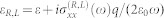, where 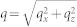 and *ε*_0_ is the permittivity of free space. For electrically isotropic 2DEGs in the absence of an external magnetic field, we have *σ*^(R,L)^_xz_ = 0 and *δσ*_xz_ = 0. Taking *δσ*_xx_ = *δσ* = *σ*^(R)^ − *σ*^(L)^, we obtain 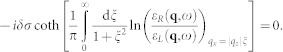
When *δσ* ≠ 0, the dispersion relation reduces to when the hyperbolic cosine of the argument in the above equation is zero. To simplify the analysis we start by setting *σ*^(R,L)^′ = 0, which neglects ohmic and interband losses (the complex-conductivity case is treated in Supplementary Information), e.g., pristine graphene in the local and zero-temperature limit^30^ at a normalised frequency Ω < 2; 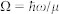, where *μ* is the Fermi energy. Then, making the variable substitution sin*θ* = (1+ξ^2^)^−1/2^, we arrive at equation (1) with the prescribed definitions. Inspection of equation (1) reveals that the left hand side of the plasmon dispersion can be written as the difference of two integrals *f*(*KN*) − *f*(*N*), where 
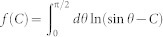
. While the solution of this integral lacks a simple closed-form expression for arbitrary *C*, its numerical solution combined with its asymptotic behaviour is revealing. We find that Im[*f*(*C*)] = 0 when *C* ≤ 0, Im[*f*(*C*)] = *π*^2^/2 when *C* ≥ 1, and Im[*f*(*C*)] increases monotonically as *C* increases within the range 0 < *C* < 1 (see the inset of Fig. 4a; dashed red and blue curve). This behaviour immediately precludes solutions for purely real *N* (i.e., non-leaky) when *K* > 0 (i.e., metal-metal systems) because 0 < *f*(*KN*), *f*(*N*) ≤ *π*^2^/2, which constrains −*π*^2^/2 < Im[*f*(*KN*) − *f*(*N*)] < *π*^2^/2, so that the imaginary part of equation (1) could not be satisfied for any integer *m*. Indeed, this was earlier pointed out by Mikhailov^27^ and corresponds to leakage of the so-called inter-edge plasmon into planar 2D plasmons towards the region with the smaller carrier density (i.e., smaller *σ*″).

Now, we turn to the pertinent case of the 1D metal-dielectric (1DMD) interface [*K* < 0; *σ*^(L)^″ > 0, *σ*^(R)^″ < 0)]. This case has not been previously considered^26^,^27^ because of the strictly positive sign of *σ*″ in conventional 2DEGs. New opportunities to realise 1DMD interfaces in hybrid 2D systems prompted us to investigate fundamental solutions to equation (1) in 2D metal-dielectric systems. Our previous observations imply non-leaky solutions to equation (1) for *K*<0 when *N* > 1 (and *m* = 0), provided that Re[*f*(−|*K|N*)] = Re[*f*(*N*)] (which satisfies the real part of equation (1)); indeed, Im[*f*(*KN*)] = 0 for *NK* < 0 (i.e., *N* > 0,*K* < 0), and Im[*f*(*N*)] = *π*^2^/2 for N > 1, thus satisfying the imaginary parts of equation (1). From the numerical integration of *f*(C), we empirically find the relation Re[*f*(*C* + Δ)] ≈ Re[*f*(−*C*)] (*C* > 0) (see the inset of Fig. 4a; overlaid dotted black curve). Setting *C* = |*K|N* leads to Re[*f*(|*K|N* + Δ)] ≈ Re[*f*(−|*K|N*)], and recalling the solution condition Re[*f*(−|*K|N*)] = Re[*f*(*N*)], we must have (comparing the *positive* arguments of the function Re[*f*(*C*)] which monotonically increases for *C* > 1, and noting Δ > 1) |*K|N* + Δ = *N*. This indicates a dispersion relation of the form *N* = *N_e_*/(1 − |*K|*), where we have identified Δ = *N_e_* as the bare edge plasmon dispersion (i.e., for when *K* = 0), or recalling the definitions of *N* and *K* and taking^28^ 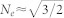 (the known approximate factor of the bare edge plasmon dispersion), we can write the following for the propagation constant: 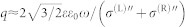 One may also consider the asymptotic behaviour in the limit *N*,*N*|*K*| ≫ 1; writing equation (1) as the difference of two integrals *f*(−|*K|N*)−*f*(*N*) and Taylor-expanding the respective arguments to the first order, we find 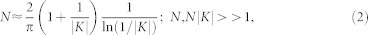
which further illustrates the limiting behaviour as |*K*| → 1 of *N* ≈ (2/*π*)(1 + |*K*|)/(1 − |*K*|), which is consistent with the (1 − |*K*|)^−1^ dependence in the empirical relation obtained above. Solution to equation (1) only when *m* = 0 naturally precludes the existence of higher-order, multipolar modes because of the step-like transition of the conductivity across the junction; this result is expected, considering that multipolar modes of a bare edge plasmon manifest only when the conductivity decreases monotonically from a constant to zero over a non-zero length *a* beyond some cutoff^21^. We note that the dispersion of the bare edge plasmon^26^,^27^ is recovered in the limit that *K* = 0, given by the root of 
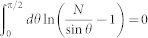
, which is satisfied by *N = N_e_* ≈ 1.217…^26^. In Fig. 4a, we plot the dispersion *N*(*K*) as determined from the numerical solution of equation (1) and as given by the analytical equation (2) given for |*K*| > 0.5 (see solid and dashed curves, respectively).

### Numerical analysis of 1DSPP in hybrid graphene/graphene system

The 1DSPP dispersion was obtained using the mode analysis tool in the COMSOL RF module (www.comsol.com). In our example of the graphene/graphene hybrid 2D system, the conductivities on either side of the junction are calculated using the random phase approximation (RPA) in the local and zero-temperature limit^30^ (Fig. 4b): 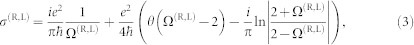
In our COMSOL simulations, the graphene is incorporated into the numerical simulations as a thin film with a thickness of *δ* = 0.2 nm, an effective relative dielectric permittivity^9^ of *ε*^(R,L)^ = 1+*i*σ^(R,L)^/*ωε*_0_*δ*, and *σ*^(R,L)^ given by equation (3). We note that the band structure of graphene is implicitly included in the permittivity through the conductivity as determined from the RPA. The almost perfect agreement between our numerical results and theoretical calculations (according to equation (1)) verify that the numerical results are sufficiently converged to the *δ* → 0 limit (at *δ* = 0.2 nm).

From our simulations, we also observed the onset of plasmon leakage towards region R (*x* > 0) as (Ω^(R)^)^−1^ >0.6, thus confirming the previous assertion^27^ for the case when *K* > 0. At values of (Ω^(R)^)^−1^ < 0.5, the dispersion must be solved for a complex value of *N* because of the introduction of non-zero *σ*^(R)^′; nevertheless, considering that we would likely have *σ*^(R)^′ > |*σ*^(R)^″| (see Fig. 4b), the 1DSPP will be strongly damped.

## Author Contributions

D. M. and S. M. contributed equally to this work. D. M. and S. M proposed and verified the existence of the 1DSPP. D. M. and S. M. performed theoretical and numerical calculations, S. Y. assisted in data interpretation, and N. P. supervised the work and added physical interpretations of the results. All authors participated in discussions and in writing the manuscript.

## Supplementary Material

Supplementary Information 1DSPP Supplementary Information
