# Supplementary material for: Plasmonic Excitations of 1D Metal-Dielectric Interfaces in 2D Systems: 1D Surface Plasmon Polaritons
Source: Sci Rep. 2014 Apr 1;4:4536. doi: 10.1038/srep04536 (PMC3971411; doi:10.1038/srep04536)
Supplement: Supplementary Information — 1DSPP Supplementary Information [file srep04536-s1.pdf]

Supplementary Information

Plasmonic Excitations of 1D Metal-Dielectric Interfaces in 2D Systems: 1D Surface Plasmon Polaritons

Daniel. R. Mason, Sergey. G. Menabde, Sunkyu Yu, and Namkyoo Park*

Photonic Systems Laboratory, School of EECS, Seoul National University, Seoul 151-744, Korea

*nkpark@snu.ac.kr

1. 1DSPPs on Hybrid Graphene 2D Systems at Finite Temperature

We investigate the propagation length of 1DSPPs in hybrid graphene systems at finite temperature. The complex conductivity of each graphene domain can be numerically calculated using the RPA in the local limit as follows[1]:

$$\sigma_{gr} = \ln\left[2\cosh\left(\frac{\mu}{2k_B T}\right)\right]\frac{2ie^2 k_B T}{\pi\hbar^2\left(\omega + i\tau^{-1}\right)} + \frac{e^2}{4\hbar}\left[H\left(\frac{\omega}{2}\right) + \frac{4i\omega}{\pi}\int_0^{+\infty}\frac{H(x) - H\left(\frac{\omega}{2}\right)}{\omega^2 - 4x^2}dx\right], \quad \text{(S1)}$$

where $$H(x) = \frac{\sinh\left(\frac{\hbar}{k_B T}x\right)}{\cosh\left(\frac{\mu}{k_B T}\right) + \cosh\left(\frac{\hbar}{k_B T}x\right)},$$

where T is the temperature, k_B is the Boltzmann constant, and $\tau = m\hbar\sqrt{\rho\pi}/(e\upsilon_F)$ is the electron-scattering relaxation time[2], with m as the mobility of graphene, ρ as the carrier concentration, and $\upsilon_F \approx 10^6$ ms^{-1} as the Fermi velocity. The complex normalised effective index of the 1DSPPs, $N = N' + iN''$, is given by the solution of the 1DSPP dispersion relation [Eq. (1) in the main text] for the complex $K = \sigma^{(R)}/\sigma^{(L)}$ obtained using (S1) at the specified Fermi energies of the respective domains, $\mu^{(R,L)}$. From the obtained value of N and from the definition $N = n/n_{2D}$ ($q = nk_0$ and $n_{2D} = i2c\varepsilon_0/\sigma^{(L)}$ (note the general form applicable to the case in which $\sigma^{(L)}$ is complex; $\sigma^{(L)''} > 0$), we obtain the complex propagation constant q, from which the propagation length is conventionally defined as $l = \mathrm{Re}(q)/[2\pi\,\mathrm{Im}(q)]$, i.e., normalised to the number of optical cycles within one exponential decay length.

In Fig. S1, we show the 1DSPP propagation length for a parameter sweep over $\mu^{(R)}$ and ω for fixed $\mu^{(L)} = 1.5\hbar\omega$ and for two different temperatures, T = 80 K (liquid nitrogen temperature; left) and T = 300 K (room temperature; right). The propagation length of the 1DSPPs (l) is primarily determined by the ratio $S = |\sigma^{(R)''}|/\sigma^{(R)'}$ and increases as this ratio increases. The ohmic losses in both regions have almost no impact on l, even for very conservative estimates of the graphene mobility. At finite temperatures, when $\mu^{(R)}/\hbar\omega < 0.5$, the plasmon is strongly absorbed because of the dominance of interband transitions in region R. The maximum value of S (at fixed $\hbar\omega$) occurs at larger values of $\mu^{(R)}/\hbar\omega$ as the temperature increases from 80 K to 300 K. Furthermore, the maximum value of S itself increases as the ratio $\mu^{(R)}/k_B T$ increases, which can be understood in terms of the decreased smearing of the graphene absorption profile ($\sigma^{(R)'}$ near $\mu^{(R)}/\hbar\omega$ = 0.5) at large Fermi energy and low temperature. Thus, an increased photon energy (at fixed $\mu^{(R)}/\hbar\omega$) corresponds to a larger value of $\mu^{(R)}/k_B T$, and the loss is reduced. We

conclude that lower temperatures and larger overall doping (i.e., higher Fermi energies of both domains) provide the optimal conditions for reducing the loss of 1DSPPs in graphene/graphene hybrid systems. As an example, we find l ~100 plasmon wavelengths at T = 80 K, $\hbar\omega$ ~0.7 eV (λ = 1.8 μm), $\mu^{(L)}$ ~1 eV, and $\mu^{(R)}$ ~0.4 eV, corresponding to a propagation length of ~2.6 μm.

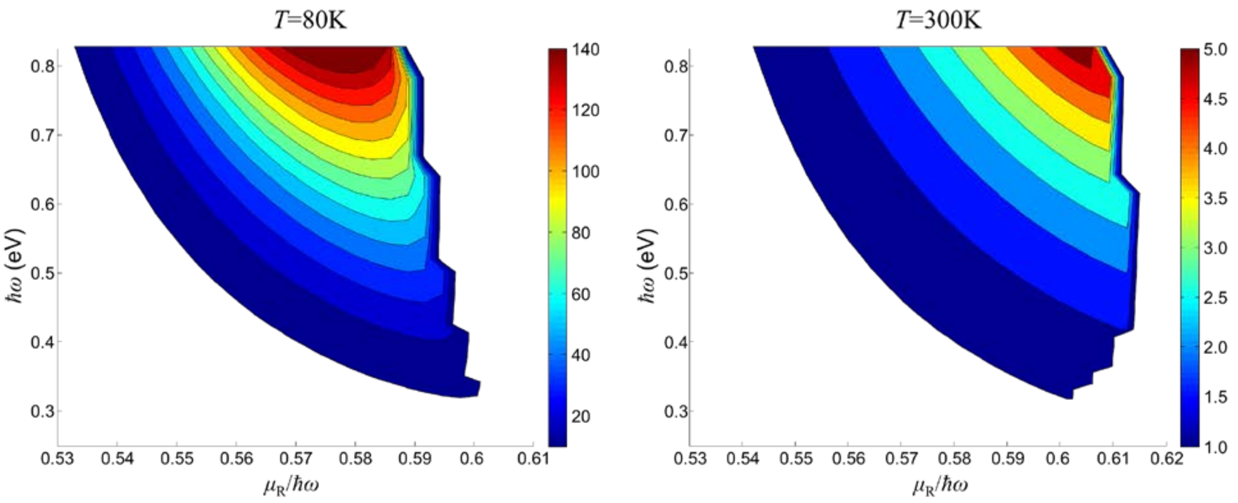

Figure S1. 1DSPP propagation length (in units of plasmonic wavelength; shown by colour contours) as a function of the photon energy $\hbar\omega$ and normalised Fermi energy $\mu^{(R)}/\hbar\omega$ of graphene in region R (x > 0). The Fermi energy in region L (x < 0) is fixed at $\mu^{(L)} = 1.5\hbar\omega$. Data are presented for two temperatures, 80 K (left) and 300 K (right). The photon energies correspond to free-space wavelengths in the range of 4 ~ 1.5 μm, and the graphene mobility is m = 1×10^4 $cm^2(Vs)^{-1}$.

2. 1DSPPs on Hybrid Graphene 2D Systems with Non-Abrupt Metal-Dielectric Junctions

Hybrid graphene/graphene 2D systems created via spatially non-uniform doping of continuous graphene sheets (for example, as proposed in Ref. 3) will exhibit a smooth transition in conductivity between the constant values $\sigma^{(R)}$ and $\sigma^{(L)}$ on either side of the metal-dielectric (MD) junction. The origin of this effect is the steady decrease in charge-carrier concentration $\rho(x)$ that occurs across the junction from the metallic domain to the dielectric domain. Thus, it is also important to understand the effects of a finite-sized conductivity-transition region on the 1DSPP dispersion when a MD interface is realised on this type of system. An important question is whether a spatially abrupt change in the sign of the imaginary part of the conductivity at the junction is critical to support 1DSPPs.

As a reasonable approximation, we have assumed a linear relaxation profile of the carrier concentration[4] across the junction (between regions L and R – see inset of Fig. S2a). The spatial dependence of the conductivity across the junction (Fig. S2a) is obtained from the RPA in the local and zero-temperature limit (equation 3 in the main text) by inserting the Fermi energy determined from the local value of the carrier concentration $\rho(x)$: $\mu(x) = \hbar\upsilon_F\sqrt{\pi\rho(x)}$. The 1DSPP effective index is then obtained from the modal analysis using COMSOL for various widths of the junction region, d. Calculations were performed for Fermi energies of $\mu^{(L)} = 2\hbar\omega$ and $\mu^{(R)}$ =

0.56ℏω at a frequency of f = 50 THz. Note that at d = 0, this MD junction corresponds to K = −0.12 and supports the 1DSPP with the third largest and asymmetric mode cross section shown in Fig. 3 of the main text.

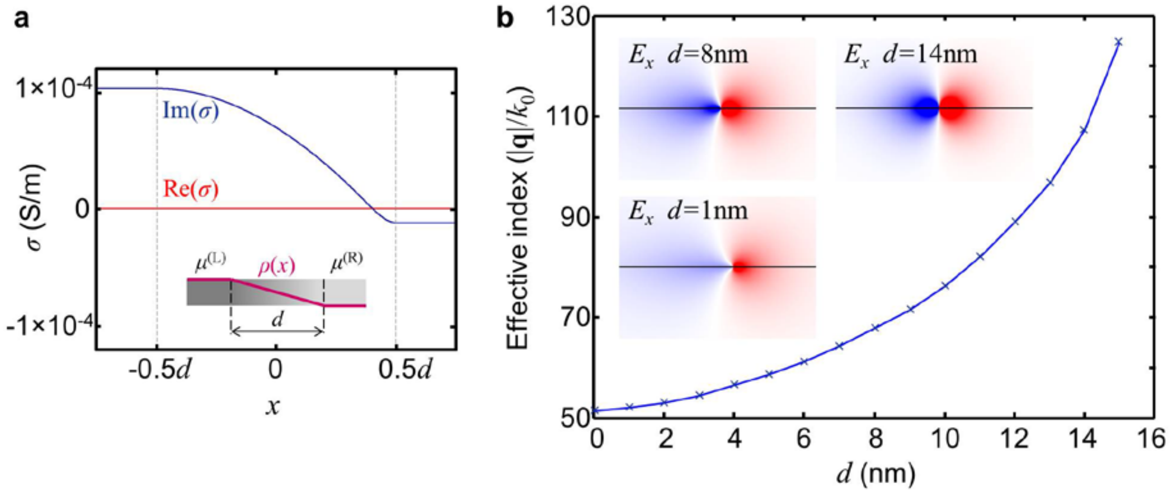

Figure S2. **a**, Spatial dependence of graphene conductivity σ across a non-abrupt MD junction of width d; the inset shows the corresponding dependence of the carrier density ρ in the linear approximation. **b**, Numerical data for the 1DSPP effective index as a function of the junction width d; the insets show the mode profiles of the electric-field component E_x at the indicated values of d. $\mu^{(L)} = 2\hbar\omega$ and $\mu^{(R)} = 0.56\hbar\omega$, and f = 50 THz.

In Fig. S2b, we show the effective index of the 1DSPP as a function of the junction width along with several sample mode profiles of the electric field component E_x. The increasingly rapid divergence of the 1DSPP effective index and the symmetrisation of the mode profile indicate that the 1DSPP may exhibit a d-dependent cutoff. It is expected that this cutoff is related to an effective local value of $K \approx -1$ (i.e., $\sigma^{(R)\prime\prime} \approx -\sigma^{(L)\prime\prime}$) near the position in the junction where $\sigma'' = 0$ [see Fig. S2a]; recall that the cutoff of the 1DSPP for the abrupt MD interface corresponds to $K \rightarrow -1$. A strong sensitivity of the 1DSPP dispersion to d is observed when the junction width is of the same order as the mode cross section of the corresponding abrupt-junction 1DSPP (i.e., with d = 0). However, the 1DSPP exists in the same form (e.g., same sign parity of all field components) with negligible perturbation when the junction width is much less than the typical dimensions of the mode cross section. In conclusion, our numerical simulations suggest that an abrupt sign change of the imaginary part of the conductivity at the junction is not critical to support the 1DSPP within the described limits.

References

1. Falkovsky, L. A. Optical properties of graphene. *Journal of Physics: Conference Series* **129**, 012004 (2008).
2. Jablan, M., Buljan, H. & Soljačić, M. Plasmonics in graphene at infrared frequencies. *Phys.Rev. B* **80**, 245435 (2009).
3. Vakil, A. & Engheta, N. Transformation optics using graphene. *Science* **332**, 1291 (2011).
4. Wang, W., Apell, P. & Kinaret, J. Edge plasmons in graphene nanostructures. *Phys. Rev. B* **84**, 085423 (2011).
